# The case of pharmacist prescribing policy in Israel

**DOI:** 10.1186/s13584-015-0045-4

**Published:** 2015-12-10

**Authors:** Hila Yariv

**Affiliations:** Poznań School of Economics, aleja Niepodległości 10, Poznań, Poland

**Keywords:** Pharmacist prescribing, Pharmaceutical policy, Pharmaceutical governance, Israel, Stakeholders, Institutions

## Abstract

Pharmacy prescribing policy in Israel has been negotiated and changed in recent years in order to improve patient treatment and access to medicines, and reduce national health insurance costs by allowing pharmacists to prescribe medications. Various stakeholders and institutions were involved in the formulation process, affecting the process while representing different motives. The complexity of pharmacy prescribing policy formulation is universal - any policy project needs, for strategic and tactical reasons, to acquire an inventory of institutions involved, identify the key players and explore potential support or opposition among them. This article uses the field (theory) of new institutional economics to explain the process of pharmaceutical institutional change and identifies the stakeholders who are involved in the reform.

In the framework of pharmaceutical policies, seven models of prescribing practices are outlined, and the Canadian and British prescribing models are presented. The paper then focuses on the Israeli case and the main issues that concern decision-makers in the Israeli health system, such as inequality in access to health services and the erosion of the notion of universal health services. These concerns and the involvement of different stakeholders, such as The Israeli Medical Association (IMA) and health funds, influenced and directed the final Pharmacist Prescribing Law. After several rejections and amendments the law was passed, enabling experienced pharmacists to prescribe only to patients with a previous prescription given by a physician in the hope it would improve services to patients and reduce physicians’ workloads. Here, the topic of the new prescribing policy is introduced, using tools from the new institutional school in political economy.

## Introduction

According to the literature in the field, governance in the health sector is difficult to define as governance operates at many different levels [1, 2]. At the broadest level, governance can be analyzed in terms of political actors who compete and collaborate to establish public policies [1]. At a secondary level, governance can be analyzed in terms of the forms of these specific public policies; the resulting rules, laws and institutions. The multilateral relationship among clients, regulators, payers and providers that exists in the health sector adds to the complexity of governance arrangements in the pharmaceutical sector [3]. The main focus of these arrangements is on transparency in the use of public funds for buying drugs, fair and equitable access to medicines, patients’ safety in the use of drugs, quality assurance throughout the supply chain and the cost effective use of drugs [3]. The United Nations proclaims that a country has good governance when its public sector acts according to principles of transparency, accountability and responsiveness [4].

Most literature regarding the governance of pharmaceuticals reviews health systems in countries that use single paid systems for their funding and where decisions related to pharmaceutical issues are made directly by their Ministries of Health [5–7]. In most countries, the profession of pharmacy is subject to professional regulation. A pharmacists’ association, a national professional organization for pharmacists, provides continual professional programs for pharmacists and keeps a register of those working in the profession. In order to practice, pharmacists must be registered with the association [8–10].

The Israeli health system is characterized by four private competing nonprofit health plans. They compete over the quality of care covered by a uniform package of benefits that is defined by the law. This package of benefits is decided by the government and financed by co-payments, an earmarked health tax as well as transfers from the general public revenues. The periodic strategic planning and development of new policies is carried out by external, highly visible, temporary commissions appointed by the Ministry of Health [11], and pharmacists do not need to be registered with any pharmaceutical association before they can practice pharmacy [12]. In terms of public health policy, Israel is heterodox as it has very high defense spending due to its potential security needs but low health spending. This normally causes a heavy constraint on the public budget and led to popular protests in 2011 [13]. In their article, Reeves and Stuckler comment on the need to recognize that the chronic underinvestment in public health in Israel is a problem [13]. This uncommon attribute adds heterogeneity to the currently existing literature in the field of governance in the pharmaceutical sector.

The main aim of this paper is to explore what factors are at play in the Pharmacist Prescribing Policy process and to find out which actors were involved in the evolution of policy making, using the field of new institutional economics to explain the process of institutional change.

Within the framework of governing the pharmaceutical sector, the focal point of this paper is the Healthcare Policy of pharmacist prescribing. This will contribute to a more complete understanding of the dynamics involved with different stakeholders.

Israelis enjoy higher life expectancy and have a much younger demographic profile than citizens of most OECD countries [14–16]. Nonetheless, the demand for healthcare is expanding rapidly due to population growth and aging, and the country’s wide socio-economic divisions are reflected in differences in health outcomes [17, 18]. Research conducted in Israel found that prescription drugs are the second health expenditure that people cut back on after dental work when facing the risk of poverty [19]. Studies conducted by Degani and Degani have shown that the proportion of individuals forced to give up prescription drugs because of their high prices was 17 % among those with a low socio-economic background, 12 % among those from the middle class and 10 % among individuals with a high socio-economic level. In addition, these studies showed that the proportion of those giving up prescription drugs was higher in the peripheral areas than in the centre of the country [20]. The findings of the research by Brammli – Greenberg et al. concluded that giving up medical treatment is a sign of a high degree of poverty. Among individuals with a low income, 20 % of the respondents had gone without medical treatment, medication or both [21, 22]. From a policy perspective, the interpretation of the relationship between socio- economic status and health implies that policies should improve access to health care, adherence to treatments, and the quality of care for patients with low socio-economic status.

The first section of this paper, Conceptualizing Stakeholders, introduces concepts related to stakeholders and institutions. The second section of the paper, Prescribing Solutions Worldwide, outlines seven models of prescribing practices identified by the writer and briefly highlights pharmacist prescribing solutions in Britain and Canada. The third section of the paper introduces the Israeli case of pharmacist prescribing policy. The fourth section discusses the achievements to date and presents future challenges.

## Conceptualizing stakeholders

This section introduces the concepts of institutions and stakeholders and casts light upon the stakeholder theory of governance, which attempts to explain how organizations can prioritize and manage relations with identified stakeholders. The section then focuses on institutions as social arrangements and concludes by describing the framework of Pharmaceutical Policy in Israel.

Governments establish the legal framework and enforce regimens that provide frameworks of actions for stakeholders and their organizations, processes that institutions have a great interest in. Scott [23] defines institutions as multifaceted, durable social structures, made up of symbolic elements, social activities and material resources. The literature of policies on healthcare systems abounds with references to institutional and stakeholder analysis, which reflects the importance of the policy making procedure and the problems that it is meant to deal with [24–26]. Indeed, any policy project, for strategic and tactical reasons, needs to acquire an inventory of institutions involved. With this purpose in mind, the writer identifies the key players involved in pharmacist prescribing policy, explores potential support or opposition among them and highlights the relevant institutions’ roles and the inter institutional linkages [27].

The term ‘prescribing’ used in this paper is as used by the National Nurse Prescribing Glossary (which was, in turn, adapted from: the National Health and Medical Research Council (1998): “the provision usually in writing by an authorized prescriber, after clinical assessment of a specified patient/client, of instructions for the dispensing or administration of medicine for that specified patient/client. Legal authority to prescribe required” [28]. Throughout this paper, the writer describes prescribing as an act that requires the knowledge of applied pharmacokinetics, adverse effects, optimal routes, doses, drug-food and drug-drug interactions, pharmacodynamics and monitoring of effects. Application of this knowledge requires significant expertise. The clinical skills that are involved in the process of prescribing include: deciding that a drug is indicated, choosing the most appropriate drug, deciding a dose and schedule appropriate for the patient’s physiological status, educating the patient about possible adverse effects, monitoring for toxicity and effectiveness and indications for seeking further consultation [29].

In this paper, the term stakeholder means groups or persons whose interests and activities strongly affect and are affected by issues concerning those who have a ‘stake’ in change, who control relevant information and resources and whose support is needed in order to implement the change [30].

A stakeholders theory addresses morals and values in managing an organization. The theory also explains and describes the web of stakeholders’ relationships that inevitably emerge within governance [31, 32]. In the case of pharmacist prescribing policy, ministers, various departments of ministries, regulators, health fund representatives, physicians, pharmacy unions, lobbyists, expert commissions and advisers interact within the official process of legislating pharmacist prescribing law. Each player hopes to advance their own agenda. Consumer health organizations and interest groups also interact under government auspices to influence health policy outcomes. This web of networking stakeholders, deliberating and negotiating, is a fundamental force needed to drive health policy reform [33, 34].

New Institutional Economics (NIE) incorporates a theory of institutions into economics. It has been developed as a movement within the social sciences, especially economics and political science [35, 36]. It unites theoretical and empirical research, examining the role of institutions in furthering or preventing economic growth. Douglass North, a professor at Washington University’s Department of Economics in St. Louis, MO, has led groundbreaking work on how institutions and societies interact and how those interactions can positively or negatively impact economics as they evolve over time [35].

North defines institutions as the humanly devised constraints that shape human interaction and calls for the analytical distinction between the rules of the game (institutions), the players of the game (individuals and organizations), and the way the game is played [35]. Pharmacy is a classic field in which to test and study NIE, as this profession is institutionally constrained by environmental factors such as medicine policies, demand for sales of over-the-counter medicines, pharmacy services provided, institutional boundaries such as the pharmacy regulation act, drug laws and regulation of drug entry and rules on exercise of the profession [37]. Consequently, the writer views the professional group of pharmacists as “players” who play to achieve better role recognition. Pharmacy is their “game”, which is played in the field of the changing healthcare environment.

In comparison to past services, the pharmacist’s services of today include more patient-oriented, administrative and public health functions [38, 39]. Therefore, the pharmacist’s role is expanding beyond the traditional product-oriented functions of dispensing and distributing medicines and health supplies. The aim of the Israeli Pharmacist Prescribing Policy, the focus of this article, is to improve patient access to medicines, making the best use of pharmacists’ clinical competencies. From a governmental point of view, objectives such as reducing waiting times for physicians, reduction in medications errors and wastage may lead to cost savings in the long run. North [40] also provides a historical perspective on the influence of different paths of institutional change on economic development. Institutional change is dependent upon (1) how different groups perceive possible opportunities and threats posed by alternative paths of institutional change or stagnation to their interests, and (2) their local, national and international political effectiveness in influencing the pace and path of institutional change*.*

### A framework of pharmaceutical policy in Israel

The term “pharmaceutical policy” describes the conscious efforts of national governments to influence the functioning of pharmaceutical subsystems [41].

Governments register medicines, compile essential medicines lists, license manufacture, procure supplies for the public sector, and dispense a substantial share of medicines through public and private facilities. In addition, they regulate prices and staff qualifications, inspect medicines for quality, collect taxes and train pharmaceutical personnel [42]. The resulting complex collection of rules, financing choices, regulatory decisions and laws constitute a nation’s pharmaceutical policy. The objectives of pharmaceutical policy vary for countries of different income levels. In low income countries, the most prevalent objective is to secure the population’s access to essential medicines. For middle income countries, the objectives are to access a broader range of medicines and to develop industry in the pharmaceutical sector [41, 42]. In high income countries, the objectives are to support the innovation of new treatments and drugs as well as provide universal access to all important treatments [42].

In Israel, drug laws are commonly issued by the Knesset (Parliament and legislative body), which also defines the rules and conditions under which the pharmaceutical sector operates [43]. The Ministry of Health (the executive branch) defines the implementation guidelines and technical standards for the law in ordinances or regulations. In common with European countries, Israel enforcement agencies and drug laws regulate the supply side parameters of the pharmaceutical market (research, quality assurance, licensing of products, promotion), while the demand side is typically regulated by legislative instruments which define who pays for which drugs and under which circumstances [44]. An example of such a legislative instrument is the Israeli Health Insurance Law. Other regulations and laws such as antitrust laws and trade laws may also influence the pharmaceutical sector. Furthermore, soft law and sectorial agreements consist a framework for pharmaceutical policy. During 2014, an updated joint treaty between the Israel Medical Association and the representative organizations of all pharmaceutical companies operating in Israel was signed. Its aim was to formulate the ethical rules underlying the professional relationship between physicians and pharmaceutical companies and to protect patients’ health and safety in this new work environment, in which extraneous considerations may overshadow scientific truth and interfere with medical decisions in the name of foreign interests [45].

Thus, policy makers need to consider these issues as well when making a policy decision. The work of expert commissions is governed in the ordinance and other relevant laws.

## Pharmacist prescribing solutions

This section records the rising costs of healthcare as well as the total volume and costs of prescribing, and presents a practical solution, namely the Pharmacist Prescribing Policy. This is followed by the introduction of seven different prescribing models that exist worldwide. The section concludes with a description of the British and Canadian pharmacist prescribing models that have been in place since 2003 and 2006, respectively.

Total prescription sales in the United States for the 12 month period ending on September 30^th^, 2013 were 326 billion U.S. Dollars, which was slightly lower than the previous 12 months (−0.7 % growth) [46]. In the U.K, prescription costs were 6199.70 million British Pounds in the 12 months between August 2012 and August 2013. Prescription volume in the same time period accounted for 740 million prescriptions [47].

The statistics portal “Statista” gives a projection of total prescription drug revenue worldwide from 2014 to 2020. In 2018, the industry is expected to generate 926 billion U.S. dollars in prescription drug revenue worldwide. This includes the leading 500 pharmaceutical and biotechnology companies and is expected to reach over one trillion U.S. dollars by 2020 [48].

There are several key issues involved in policymakers’ concerns about the viability of our healthcare systems today. These include the increased cost of new pharmaceuticals and other evolving technologies, the growing needs of aging populations, the impact of chronic diseases, and a significant workforce crisis [49]. Solutions to these issues will not come easily; however, what is clear internationally is that pharmacists, who are viewed as drug experts, can become key participants in the management of health care costs through their contribution to the informed and appropriate use of medications in community, hospital and nursing home settings [49, 50]. Pharmacists’ involvement in optimizing drug therapy is shown to prevent hospitalisation by identifying inappropriate medication use in older adults, by improving anticoagulation outcomes in pharmacist led anticoagulation clinics and by promoting medication adherence with a reduction in cardiovascular risk factors in pharmacy education programs [51].

Starting in the 19^th^ century, governments reacted to concerns about drug misuse and public safety by using medicine regulation legislation. In the early part of the 20^th^ century, prescriptive authority was limited in legislation to vets, dentists and physicians. In the latter part of the same century, a vast range of medicines on the market was used for prophylactic and treatment purposes. The scope for treatment was expanded and the focus shifted to methods of funding health while assuring timely and safe access to medications. One of the ways to deliver this was to expand the prescribing authority of health care professionals other than physicians [52].

### Models of prescribing

A review of the international pharmacy literature identifies seven models of prescribing practice. These demonstrate the potential breadth of practice and the capacity of pharmacists to initiate modify and monitor prescription medicine use with varying levels of autonomy.

### Independent prescribing by pharmacists

Independent prescribing by pharmacists occurs when the prescribing practitioner, who is a pharmacist, is solely responsible for patient assessment, diagnosis and clinical management. This position requires legally defined levels of knowledge and skills that are usually monitored through a licensing process. This model is currently implemented in Australia as well as in the UK [39, 53].

### Dependent prescribing models: prescribing by protocol

‘Dependent’ prescribing incorporates further restrictions on prescribing activities, via protocols or formularies. Prescribing by protocol is the most common form of dependent prescribing, and is defined as delegation of authority from an independent prescribing professional, usually a physician [54]. The protocol is a written guideline that describes the activities pharmacists may perform in their prescriptive authority^1^ . The protocol details the procedure plan that the pharmacist must follow when prescribing, as well as the types of diseases, drug categories, the responsibilities of each of the parties involved, and prescriptive decisions covered by the agreement [54]. Studies propose that prescribing by protocol improves access to medicines as patients do not need to visit their physician and also reduces drug costs as the prescriber is restricted to prescribing according to a specified treatment protocol, based on available evidence and for patients with a particular diagnosis [55, 56].

### Dependent prescribing models: Patient Group Directions

A Patient Group Direction (PGD) is a written direction signed by a doctor or a dentist, as well as by a pharmacist, relating only to supply and administration of prescription medicine. The PGD applies if a number of specified requirements are met and is subject to any specific exclusion listed. Only specific drugs listed in the PGD are allowed to be prescribed [55]. This policy has been implemented for the prescribing of hepatitis vaccinations for drug misusers in the UK [57].

### Dependent prescribing models: prescribing by formulary

In formulary-based prescribing, local formularies are set between participating community pharmacies and medical practitioners. The formulary includes a limited list of medicines and the length of treatment, treatable symptoms, criteria for referrals and limitations on prescribing. Considerable record-keeping is needed as well as an extra pharmacist and private consultation area [55]. This is currently implemented in Florida for a skin patch which is used to prevent nausea and vomiting caused by motion sickness [58].

### Dependent prescribing models: repeat prescribing by pharmacist

Repeat prescribing by pharmacist involves pharmacists providing medication-refill services in clinics associated with medical centres, for patients who have exhausted their prescribed drugs before their next physician appointment [59, 60]. There are few optional services that are available according to this model. According to one optional service, the pharmacist examines the patient and the therapy and then either refills the medication with a sufficient quantity to last until the next available appointment or consults the attending physician if there are problems with compliance or side effects [60]. According to a second option, a pharmacist can prescribe a further supply of medicines that were initially prescribed by a physician after conducting an effective patient – centred consultation and being satisfied that the medicines are safe and effective. Often, there are restrictions in terms of the number of prescriptions per patients that are allowed to be prescribed by the pharmacist and regulations may restrict some types of drugs from being prescribed. In line with this prescribing service, the pharmacist is required to review patients’ current treatments and conditions, and use his clinical judgment and effective consultation skills to decide whether it is safe or not to provide the patient with a prescription for the continuous supply of his/her medicines. The implementation of this prescribing model is underway in Israel and already exists in Australia [61]. The main value of this model is that it provides improved access to medicines, using pharmacist knowledge to evaluate the appropriateness of existing medications and providing continuity of care for patients with chronic diseases. The disadvantage of this model is that currently in Israel, only pharmacists who work in health funds pharmacies are able to access patient medical records, whilst pharmacists working in community chain and privately owned pharmacies, are unable to access these records.

### Dependent prescribing models: supplementary prescribing

Supplementary prescribing, is a voluntary partnership between the independent prescriber and a supplementary prescriber, to implement an agreed patient-specific clinical management plan (CMP) with the patient’s agreement [62]. The independent prescribers are physicians or dentists who undertake the initial assessment and the supplementary prescribers are registered pharmacists or nurses who then write the prescriptions [63]. The CMP provides detailed guidance for each stage in the management of a patient with a specific condition over a given time period, and includes progress and outcomes details. This type of prescribing is tailored to patient’s needs and is therefore believed to improve clinical outcomes and help reduce costs by shortening hospital stays due to comprehensive medication reviews with the pharmacist that contributes to increased patients compliance and improved patient safety. A further benefit of the model is the improvement in multidisciplinary communication, teamwork and care planning between health sectors [59]*.*

### Collaborative prescribing models

Collaborative prescribing requires a cooperative practice relationship between a pharmacist and a physician or practice group, with legal authority to prescribe medications. Explicit collaborative agreements are negotiated within each facility, outlining who is receiving authority and delegating, and a demonstration of competence. Firstly, the physician diagnoses and makes initial treatment decisions for the patient, and secondly selects, initiates, monitors, modifies and continues or discontinues pharmacotherapy as appropriate to achieve the agreed patient outcomes. The physician and pharmacist share the risk and responsibility for the patient outcomes. Collaborative prescribing is used in several countries such as France, Switzerland, and almost all states in the US [64].

This next section follows reports from the American Pharmacist Association and focuses on two countries, Britain and Canada, where the healthcare innovation of extending prescribing authority was driven by different factors [65]. In Britain, the driving force was the need to improve services to vulnerable groups such as the elderly and the disabled. In Canada, the driving factor for pharmacist prescribing was the shortage of physicians in remote areas [65]. Israel has longevity in common with Britain and a shortage of physicians in peripheral areas in common with Canada. According to the Central Bureau of Statistics (CBS) population projections, the elderly population in Israel is expected to reach 1.367 million in 2030 – a 84 % increase on the 2009 figure [66].

The introduction of a pharmacist prescribing policy provides a new facet of improved accessibility to elderly patients in primary care. The Israel Medical Association reports that there is a disparity between the scopes of healthcare services available in the periphery compared to central Israel. One of the most complex problems with health services in the periphery is the difficulty in attracting medical and nursing manpower from central Israel [67]. As pharmacists are available for consultation when other care providers are geographically inaccessible, pharmacist prescribing has great potential to immensely improve the health of Israelis. Therefore, pharmacists can play a role in eliminating and addressing health disparities.

### Pharmacist prescribing model in Britain

There are two models of pharmacist prescribing in Britain. Pharmacist Supplementary Prescribing (SP) was introduced in 2003 and involves a voluntary partnership between the responsible independent prescriber (a physician or a dentist), the supplementary prescriber (most commonly a pharmacist or a nurse) and the patient. As part of the prescribing process there is a need to implement an agreed patient-specific clinical management plan (CMP) [10, 62]. Time spent initially developing a simple CMP eventually saves time when the patient returns for review to the supplementary prescriber rather than to the physician. Pharmacist independent prescribing (IP) was introduced in 2006. In this model, the pharmacist is the sole prescriber responsible for the assessment and consequent management of the patient’s condition [62].

The legal basis of supplementary prescribing was the Health and Social Care Act of 2001 which enabled the government to extend prescribing responsibilities to other health professions. Amendments to the Prescription Only Medicines Order and NHS regulations allowed supplementary prescribing by suitably trained nurses and pharmacists from April 2003 and podiatrists, physiotherapists and radiographers from 2005 [68]. The objective of the model was to provide patients with quicker and more efficient access to medicines while making the best use of the clinical skills of eligible professionals. In the government’s view, the aim of the policy was to reduce doctors’ workloads, giving better opportunity to tend to patients with complicated conditions and complex treatments [62].

The main facet of the model is maintaining communication between the independent and supplementary prescribers, allowing them to consult, update, share access to the same local or national guidelines or protocols, agree and share a common understanding of and access to the written CMP [63].

In terms of education, a pharmacist who is trained to become a supplementary prescriber is asked to undertake a specific training program at degree level. The program comprises approximately 25 taught days plus at least 12 days ‘learning in practice’ [53].

At first, the formulation and implementation of Pharmacist Supplementary Prescribing was met with strong opposition from the medical community. The World Medical Association added that certain tasks can only be performed by physicians, prescribing being one of those things [63]. However, the program is now considered to be well-integrated and to have greatly contributed to patient care which is safe and of good quality [53, 69].

### Pharmacist prescribing model in Canada

Traditionally in Canada, the authority to prescribe medications has rested with a small number of professions. With changes to legislation or regulations in most Canadian provinces over the past eight years, many pharmacists now have the ability to initiate, continue or modify drug therapy, ranging from renewing a continuous care prescription to independent prescriptive authority [70]. In 2013, community pharmacists prescribed for flood victims in Calgary who had to evacuate their homes in the middle of the night, leaving their medications behind [65].

The Canadian Association of Pharmacist Prescribing reports that medication adherence is particularly a challenge for the treatment of chronic conditions in Canada. As most provinces limit prescription length to three months, four million Canadians report not having a regular physician nor access to primary care doctors for the purpose of their prescription renewal. The Association supports the granting of pharmacist prescribing authority to improve medication adherence by making refills and emergency supplies more readily available to these patients [70].

A comprehensive policy review and comparison of documents and regulations written by the relevant government and professional agencies in Canada shows that in terms of pharmacist prescribing, legislative or regulatory enactment of current privileges are in place or expected in the future across different provinces [65, 70]. Findings show that substantial variation exists in the scope of pharmacy practice across provinces as provincial pharmacy regulatory bodies vary in governance structure, legislation and standards and codes of conduct [54]. Overall, prescribing policies have three typical forms: firstly, allowing pharmacists to renew prescriptions for long-term conditions, secondly, permitting short-term dispensing to allow patients to continue therapies uninterrupted and thirdly, allowing pharmacists to prescribe in emergency situations. In addition, existing provincial policies also vary in the type of formal and experiential education required of prescribing pharmacists as well as in terms of knowledge, skills and continuous professional development [50, 70].

To that end, Israeli policy-makers can learn from the cases of these two countries and ascertain the determinants of success in obtaining pharmacist prescribing reform in Israel.

An integrative review of literature on non-medical prescribing in primary care undertaken by Bhanbhro et al. [59] shows that in the 21^st^ century non-medical prescribing has evolved differently in different countries. The study demonstrates that out of the 193 countries that are member states of the World Health Organization (WHO), twenty countries provide legal authority to nurses and other health professionals to prescribe medications, while other countries are considering introducing legislation. With respect to stakeholders’ acceptance of the new pharmacist prescribing role, studies show that health professionals and patients widely approve and view positively the non-medical prescribing initiative [39, 59, 71].

Five models for Pharmacist Prescribing have been identified and deemed suitable by the Israeli Knesset during the policy formulation stage: repeat prescribing model, prescribing for minor illness model, emergency prescribing model, prescribing by protocol and independent prescribing [72]. After much deliberation in the Knesset, it was decided that, initially, the only prescribing to be put into practice would be the repeat prescribing model. According to this model, during a prescribing consultation, the pharmacist interviews, listens and examines the patient’s medical records, then checks the current drug therapy to decide whether it is safe to issue a prescription with a further supply of medicines, that were initially prescribed by a physician, or refers him back to his physician for a further consultation. The IMA shared an objection to the rest of the models, thence most were postponed indefinitely. However, the Knesset’s chairman and the head of the Health Ministry agreed that the prescribing for minor illness (such as coughs, allergic reactions, sunburn and skin infections) would be revisited once the implementation of the repeat prescribing model was complete [73].

## The Israeli case

This section unfolds the story of the pharmacist prescribing initiative in Israel. It begins by pointing out the essentiality of the policy to the Israeli health system and continues by revealing the process of agenda setting. The section then describes the chain of events that resulted in policy formulation and identifies the key objectors to the policy.

To date, the Israeli health-care system, centered on four health funds (to the tune of 95 % of the total market), is widely acknowledged as providing a “package” of universal services of good quality primary and secondary care, while also accommodating demand for private health care [74, 75]. Nevertheless, there are challenges and tensions in the system. In recent years, the authorities have worked to expand the number of students trained in medical schools and nursing training as large cohorts of healthcare professionals are heading for retirement [76]. Media reports and public opinion polls show that Israelis are dissatisfied with the amount of time people wait to receive medical care [77, 78]. As a consequence, the demand for health care services exceeds the supply of those services and some measures must be taken to allow better access to health care. An example of such a measure, exploiting opportunities to shift tasks from physicians to pharmacists, is the case of the Pharmacist Prescribing Initiative.

Entrusting pharmacists with more responsibilities is likely to reduce current demand on other, more expensive health care resources such as hospital emergency rooms and physicians. A study investigating pharmacists’ perceptions of the value of pharmacist prescribing of antimicrobials in hospital settings in Scotland found that “optimization of antimicrobial use through pharmacist prescribing of antimicrobials was also perceived as indirectly leading to a reduction in overall costs through shorter bed stays, using cheaper but same-spectrum antimicrobials, ensuring appropriate duration and switching to oral use when feasible” [79].

Concerns about strict practice policies and liability associated with prescribing are often reported. A research article describes that to resolve these concerns, pharmacists tend to reduce the amount they prescribed, take care to document and take additional time to review clinical information [80]. This tight control is hoped to eventually result in lower costs to the health system and better health outcomes for individuals.

In Israel, the potential healthcare cost savings were also a driving force for the adoption of the policy [73]. As pharmacists are generally on lower wages than physicians, the 2014 BMI report estimates that shifting and reducing labor costs means that funds will be released for spending on other areas of the healthcare system. The report foretells that prescription drug sales in 2014 are expected to amount to 1.68 billion U.S. Dollars, a 7.72 % increase on 2013. The report further foretells that due to favorable epidemiological factors in Israel, such as an aging population and population growth, continued increase in demand for drugs for chronic diseases and physicians’ consultations is expected [14].

In July 2014, after a decade of negotiation and deliberation, the regulations for the Pharmacist Prescribing Law in Israel were finalized and the ordinance was published. This policy is much needed as recent data shows that income inequalities in Israel are wide and persisting. The average income of the richest 10 % of the population in Israel is about 14 times that of the poorest 10 % [81]. The Gini coefficient for Israel, which is a measure of income inequality ranging from zero (full equality) to 1 (when only one person concentrates all income), is among the highest in the OECD [82]. The rise in private funding has been found to affect patients’ compliance and concordance with their medications [83, 84]. The pharmacist prescribing initiative aims to improve adherence and contribute to achieving better health outcomes for Israeli patients.

The 2014 Business Monitor International paper reports that those on the lowest incomes in Israel struggle to pay for medical services fully, despite the well-functioning universal health system [14]. As rural areas often have a greater demographic need, research has shown that there is a link between health disparities and rural living [36]. An OECD review of the tackling of inequalities in health in Israel reports that people who live in the periphery have worse health indicators than people who live in urban areas [85]. A more recently published OECD report highlights the wide variations in healthcare in Israel. The findings suggest that either unnecessary care is being delivered in areas of urban living, or that there is unmet need in rural regions [86]. Thus, local pharmacists are particularly valuable assets in impoverished urban areas. The Pharmacist Prescribing healthcare policy aims to resolve these issues as will be outlined in this article.

Studies have shown that creating channels for stakeholder participation in managing or supervising public healthcare services can improve the performance of services in several ways [27, 36]. One example is the opening of policy decisions in Israel to professional associations to protect health professionals’ interests and safeguard the medical quality of care. Since Israel does not have a comprehensive national health plan or an active system for setting and updating national health targets, periodic strategic planning and development of new policies are carried out by external, highly visible, temporary commissions appointed by the Ministry of Health [11]. In his report, Rosen names several Israeli health-related commissions and adds that due to the Ministry of Health’s multiplicity of roles, the latter’s commissions are perceived as being able to examine issues related to healthcare closely, efficiently and objectively [11].

The journey towards extending pharmacists’ responsibilities began in 2000 when the National Board to promote the pharmacy profession, named the Benita Board, was commissioned by the Ministry of Health with the aim of discussing the future of the pharmacy profession in Israel. The board was the first to introduce the idea of extending pharmacists’ responsibilities by giving pharmacist prescribing authority [87]. To make this happen, an amendment to the existing Pharmacists’ Ordinance (new version - 1981) had to be made.

In 2006, the Ministry of Finance’s proposal sought to amend the Pharmacists’ Ordinance (new version – 1981) so that it would enable a pharmacist to issue a prescription drug not based on a prescription signed by a physician, and to amend the Physicians’ Ordinance (new version – 1976), so that the authority of the Director General of the Ministry of Health to permit individuals who are not physicians to perform extraordinary medical procedures will be expanded to para-medical professionals [88]. It was decided that a joint committee of the Ministry of Finance and the Ministry of Health would be established with the aim of determining which medical activities, currently performed only by doctors, would be authorized for pharmacists, nurses or para-medical professionals. The Ministry of Finance believed that the policy would save physicians’ time and reduces pressure through sharing the patient load. The emerging data at the time, which compared the cost of pharmacist prescribing to physician prescribing, demonstrated that one of the benefits of the scheme was that pharmacist prescribing proved to be more cost effective [39, 59]. The Golomb Committee was commissioned to find possible ways to assimilate the amendment to the Pharmacists’ Ordinance and to suggest possible ways to regulate prescribing as well as dispensing medicines not in accordance with physician prescription. Within a short period of time, a new ordinance was suggested to allow pharmacists to prescribe, but it has since been invalidated by the Justice department by the claim that it diverges from the provision of the present law and creates a new professional entity not defined by it. As a result, The Knesset rejected the ordinance [89].

In 2009, the Pharmacists’ Ordinance was amended once again. This time it was passed successfully in the Knesset as part of The Arrangements Law, a government bill that is presented to the Knesset each year alongside the Budget Law. It incorporates government bills and legislative amendments that are needed in order for the government to fulfill its economic policy [90]. The Arrangements Law is a unique instrument used by the government to initiate legislation, complete legislative acts and stall or eliminate private members’ bills already being legislated. In essence, through the law, the government can overcome parliamentary obstacles, as it does not need to be approved by the various committees, as ordinary bills usually do. It was not until 2011 that the amendment to the existing law was approved. During 2011, the advocates of the Pharmacist Prescribing Policy encountered a further obstacle raised by a second stakeholder, namely the Israeli Law, Information and Technology Authority (ILITA). The ILITA was established by the Ministry of Justice to become Israel’s data protection authority. After much debate between the ILITA, the Pharmaceutical Society of Israel and members of the Ministry of Health, a new version of the law with an amendment concerning the protection of data shared during the prescribing process was introduced [91].

In 2014, the Pharmacist Prescribing Policy was finalized and the regulations that will enable pharmacists to prescribe were approved. The ordinance specifies various conditions for prescribing to take place, four of which are listed here. *Firstly,* it was decided that in the first stage of implementation, pharmacists will only be able to prescribe medications for chronic conditions that have been previously prescribed by a physician (i.e. the repeat prescription model as outlined previously). Many stakeholders were disappointed to discover that only one out of the original five models was approved by the Knesset. *Secondly*, it was determined that only pharmacists with a minimum of five years’ experience will be allowed to prescribe for a list of conditions such as diabetes, Parkinson’s disease, muscle pain, thyroid disorders and urological problems. The law allows pharmacists to issue a repeat prescription only up to six months after the expiration of the original prescription. *Thirdly*, it was agreed that pharmacists will have to complete an eighty-hour course and pass an exam before being able to prescribe [92]. The first prescribing course commenced in January 2015 [93]. Finally, to support optimal medication management, pharmacists require access to patient medical records during a prescribing consultation. The rationale behind this is that prescribing should also involve an accurate, legible and comprehensive written prescription based on looking at a patient’s drug history, accessing previous lab results and documenting the consultation in the patient’s records to establish the continuity of care. At present, patient medical records can only be accessed by pharmacies that belong to the health funds. Pharmacists who work in privately owned pharmacies are unable to access those records.

In an interview with Mednet, The Director of the Professional Committee of the Pharmaceutical Society of Israel, Dr. Ron Tomer, commented that the aim of the policy is to improve patients’ service in terms of quality and availability of treatment, reduce physicians’ workloads and save on health expenses. Dr. Tomer explained that the extension of prescribing rights will start with pharmacists only renewing prescription drugs, being responsible for the continuing care of patients who have been clinically assessed by a physician. “Eventually, one hopes”, he said “that suitably qualified pharmacists could become independent prescribers in their own right” [94].

Records show that the four health funds supported the policy throughout the implementation stage [43]. One may speculate that one of their motives was the future cost savings involved due to the reduction in labor costs. Finally, the shift in prescribing policy means that physicians will have additional time to attend to more important issues in the six months interval between patient visits.

The Israeli Medical Association (IMA) was an influential stakeholder that opposed the Pharmacist Prescribing Policy throughout, arguing that the prescribing policy lacks budget ramifications and is therefore not related to economic policy – yet it was passed through the Arrangements Law, a framework whose sole aim is to deal with budgetary and economic aspects [89]. The members commented that the issue of transferring medical authority is complex and has fundamental ramifications for the state health system and for the condition of patients and therefore should only be accepted if treated by the Knesset as a standard bill which is usually advanced in a number of stages (“readings”) until it is finally passed [89].

The patient advocacy organization was involved in the initial stage of the legislation. Their concerns involved securing patient confidentiality and allowing patients the choice to decide whether to use the service or not. Once these two issues were cleared up (patient confidentiality was defined in the law and it was clear that the service was optional), the organization ceased to be involved.^2^

Overall, the Pharmacist Prescribing Law was legislated twice and the legislation process lasted nearly a decade, mainly due to the opposition of various stakeholders who were mentioned in this article. The leading argument of the IMA was the state’s commitment to the Patients’ Rights Law that declares the right of every patient to receive good medical treatment with respect to the professional level and quality of medicine [89]. According to them, the logic underlying the singularity of medical practice according to the Physicians’ Ordinance can be expanded to the authority of others and may therefore contradict the legal standard. They claimed that there is no substitute for the extensive years of study and enormous professional knowledge accumulated by physicians in diagnosing and providing medical treatment to the patient and those health professionals who are not physicians are not as likely to be able to remain up-to-date with the latest scientific literature. As mentioned earlier, the Independent Prescribing model was withdrawn from the final stage of deliberation due to the raised objection by the IMA, which has powerful and influential status in the Knesset [73]. This was a big setback for pharmacists.

The law was also disputed by the Labor and Welfare Committee, which on several occasions protested against the passing of the law through the hurried Arrangement Law system and claimed that a much longer debate was needed. Nonetheless, the Knesset protocols provide evidence that on several occasions during the voting process, the majority of the committee members voted in favor of the policy [73].

When interviewed by the writer, a few different stakeholders questioned the necessity of the pharmacist prescribing service due to the fact that nowadays physicians are allowed to prescribe “refill authorizations” that provide patients with access to medications until their next medical visit. Their opinion was that most patients order their prescriptions online, making the pharmacist prescribing service, again, unnecessary. They claimed that ten years have passed since the commencement of pharmacist prescribing policy legislation and meanwhile, with the advance of the internet and the online medication ordering service, the policy has become redundant.^3^ Another, relatively new technology in Israel is e-prescribing (also called electronic prescribing) that was first introduced in 2010. This is a technology framework that allows physicians to write and send prescriptions to a participating pharmacy electronically. The 2010 Milken Institute report explains that “ in terms of CPOE (computerized physician order entry; the process of a medical professional entering medication orders or other physician instructions electronically) and e-prescribing, Israeli usage is 95 percent” in comparison to the United States, “where their usage is just 20 percent” [95]. Online medication ordering and e-prescribing services are estimated to save the Israeli economy over 11 million physician working hours per year. It is possible that what stakeholders considered as an essential service in the past (i.e. pharmacist prescribing) has become non-essential with the advance in technology.

There is a consensus among the interviewed stakeholders about the need for additional pharmacist training and staffing to fulfill their new prescribing role as well as a properly aligned incentive to compensate for these additional professional costs and for the additional value brought to the citizens of Israel. To date, no such financial incentive exists.^4^

There was no involvement of the Israeli Pharmaceutical Industry during the legislation process as generic substitution at the point of dispensing in both private and public sector facilities is allowed. This is in contrast to the U.S., where one of the main barriers to the Pharmacist Prescribing Initiative was the pharmaceutical industry’s objection to the policy, since they perceived pharmacists as being more likely to prescribe cheap generics than physicians, which will damage their sales [54].

## Discussion

This article highlights recent changes to the pharmacy regulations that will empower pharmacists to provide prescription medications to better serve Israelis. The Israeli patients who are most likely to benefit from the new regulations are those who suffer from chronic conditions, such as diabetes and asthma. The aim of the policy is to provide seamless, faster care to patients to obtain their medicines through making better use of pharmacists’ skills. Hopefully, these regulations will improve access to medical services and the efficiency of the health system overall [14]. Deputy Health Minister Ya’akov Litzman expects that these regulations will allow pharmacists to fully utilize their expertise in medication management in the interests of the wellbeing and health of Israelis, particularly those who live in the periphery (suburbs) [87].

Stakeholders’ views on the expected economic impact of pharmacy prescribing vary.

It is expected that the introduction of the service will lead to cost saving in the healthcare system through reduction in unnecessary medication use and increased patient choice in accessing medicines. An analysis to measure the impact on patient health outcomes and cost benefit analysis is required as this prescribing model rolls out. These results might be key factors at play in the Pharmacist Prescribing Policy process.

The IMA’s response to the policy can be viewed, within the framework of challenges to medical dominance, as a logical reaction to the potential loss of professional status. Mesler [96] notes that when some professionals [e.g. pharmacists] obtain and deploy resources, others [e.g. physicians] do not necessarily lose them. Finally, it is envisioned that through a multi-disciplinary and collaborative approach, expanding pharmacists’ authority to prescribe will help to achieve an optimal public health outcome.

The extent to which the Pharmacist Prescribing Policy will be implemented successfully depends on current policy imperatives. For instance, in 1997 the Department of Health in South Africa withdrew pharmacists’ permits that enabled them to provide prescription only medicines at their discretion [97, 98]. In Canada there have been several attempts to create system wide pharmacist practice change before any reform is successful [54].

Currently, there are no mechanisms by which community pharmacies can be reimbursed for the prescribing service. Data gathered from interviews with key Israeli stakeholders suggests that in their opinion, this challenge is the biggest one to yet be overcome. Furthermore, critics of this advance in the practice of pharmacy will not be reassured that, under the proposed standards, it will be acceptable for a prescribing pharmacist to also dispense the medicine that he prescribed to the patient. The lack of separation between prescribing and dispensing questions the pharmacists’ ability to ensure patient safety and prevent any potential conflict of interest. If pharmacists are only compensated for dispensing medications then this may lead to the possibility that pharmacist prescribing has an inherent conflict of interest for pharmacists. They would be inclined to prescribe medications (instead of eliminating unnecessary medications) so that they could sell more drugs.

Several stakeholders were concerned about the lack of an insurance provider who is able to provide professional indemnity cover for the extended role of prescribing.

The need for pharmacists’ education is an additional hurdle. Today, the prescribing course is relatively expensive, lasts for six months and entails passing an exam to qualify as a pharmacist prescriber. When interviewed, two official stakeholders from the health funds explained that they currently have no funds to send their pharmacists on the training course. On a positive note, one hundred pharmacists attended the first prescribing course that commenced in January 2015. An unpublished study conducted by the writer investigates the opinions of the course participants regarding the benefits, challenges, facilities and concerns regarding the introduction of the pharmacist prescribing policy. The preliminary results show that the increase in job satisfaction, elevated professional status and better use of pharmacists’ skills were the most important factors when deciding to become a prescriber. When asked about concerns they may have regarding the implementation of the policy, pharmacists stated that access to patient medical records and documentation of the consultation were paramount. Coping with heavy workloads and staffing issues to clear time for consultations and keeping up to date are additional challenges prescribing pharmacists have to face. Thus to overcome the barriers to prescribing, there needs to be more support (financial and logistical) given to pharmacists who wish to prescribe. In order to resolve resistance from other prescribers and eliminate the potential for boundary conflicts, there is a need for a clarification of the pharmacist prescriber’s role and the fostering of collaborative relationships to allow smooth integration of pharmacist prescribing into practice.

It is not possible to map out in advance an optimal and desirable path for the policy process, and the path is decided by political processes and tradeoffs between different stakeholder groups as they try to achieve a change or to preserve the institutional environment and institutional arrangements in order to suit their interests and advance their own agendas [99]. Thus, an understanding of the potential roles of the stakeholders and the institutions involved in order to identify a potential coalition of support for the policy is paramount for the success of the policy’s implementation and evaluation. Achieving a system-wide practice change is a challenge that the profession of pharmacy continues to face.

## Footnotes

1 Occasionally, the first two models co-exist. For example, in New Mexico, pharmacists are primarily independent prescribers. An exception to this is the “Naloxone rescue kit”, which pharmacists can prescribe dependently. In Washington State, there are thousands of different protocol-based prescribed therapies, one of which is the emergency contraceptive pill [59, 100]. There are now at least 46 U.S. states that allow prescribing by protocol and several states that allow at least some degree of independent prescribing [for this additional information I would like to thank the blind reviewer].

2 Though there is no direct, explicit mandate for involving patients in decisions relevant to their health in Israeli law, the requisite conditions are encapsulated in the Patients’ Rights Law of 1996.

3 Personal interviews, Jan-March 2015. All interviews were confidential; the names of the interviewees are withheld by mutual agreement

4 Personal interviews, Jan-March 2015. All interviews were confidential; the names of the interviewees are withheld by mutual agreement.
